# Educational design insights for interprofessional immersive simulation to prepare allied health students for clinical placements

**DOI:** 10.1186/s41077-024-00316-0

**Published:** 2024-11-27

**Authors:** Jennie Brentnall, Laura Rossiter, Belinda Judd, Emma Cowley, Keith McCormick, Ruth Turk, Debbie Thackray

**Affiliations:** 1 University of Sydney, Sydney, NSW 2006 Australia; 2 University of Southampton, Southampton, SO17 1BJ England

**Keywords:** Allied health, Interprofessional learning, Practice learning preparation, Simulation-based education, Clinical placements, Work-integrated learning

## Abstract

**Background:**

Positive outcomes of simulation programmes to prepare students for placement are widely noted. However, few studies adequately describe considerations for designing allied health placement simulations. There exists a conceptual framework to guide such simulation design, which draws on theory and educational expertise but to date lacks varied stakeholder perspectives. This study aimed to identify implications for the design of allied health placement simulation from participants’ experiences of a simulation-based, interprofessional, novice placement preparation programme.

**Methods:**

Occupational therapy, physiotherapy and podiatry students finishing their first year of study were offered a 1-week intensive interprofessional simulation immediately before their first placement. Focus groups in the following weeks allowed participants to discuss their experiences of the programme, preparation for student placements, and recommendations. These were transcribed and interpreted using reflexive thematic analysis and then abductively related to the conceptual framework.

**Results:**

In total, 22 participants broadly representative of the simulation programme participants contributed to separate focus groups with domestic-enrolled students (*n* = 7), international students (*n* = 5), external practice educators (*n* = 6), and simulated participants (*n* = 4). Inductive reflexive thematic analysis generated six themes: (i) engaging learning environment, (ii) realism and relevance, (iii) student confidence and communication, (iv) international students’ needs, (v) recommendations to facilitate further preparation for placement, and (vi) importance of preparation to engage in simulation.

All participant groups were invested in the programme and highlighted learning opportunities. An immersive and relatable experience with active participation contributed to confidence and communication skill development. International students noted needs pertaining to cultural and language expectations. Other participant recommendations related to the equity of opportunities and specific preparation for placement student-educator interactions. Finally, every participant group noted features of effective preparation for simulation-based education.

**Conclusions:**

Relating these findings abductively to the literature and conceptual frameworks, this study highlights simulation design considerations for learner needs assessment, engagement, realism, psychological safety, and challenge to prepare learners for their first clinical placement. Specific implications for adequately preparing all participant groups, design considerations for the needs of culturally diverse students, and balancing interprofessional and discipline-specific learning are highlighted from a lived experience viewpoint. Future research may engage greater stakeholder co-design in simulation-based education.

**Supplementary Information:**

The online version contains supplementary material available at 10.1186/s41077-024-00316-0.

## Article

Educational strategies informed by evidence from multiple stakeholder perspectives are needed to ensure allied health students are adequately prepared and supported to successfully transition to learning in clinical placement environments. This study focuses on the evidence-based design of simulation-based education approaches that prepare students for novice clinical placements by preceding and partially replicating those placements. The application and effectiveness of simulation immediately prior to placements are well-established, but the theoretical frameworks and factors informing the design of these experiences are infrequently described [1]. The Conceptual Framework for Simulated Clinical Placement (CF-SCP) was developed to guide simulation design in such contexts, drawing on simulation and learning theories and expert opinion [2]. Taking a pragmatist approach, in this study we apply the CF-SCP and draw specifically on the perspectives of international- and domestic-enrolled students, clinical educators, and actor role players, as well as clinical practice, curriculum, and simulation expertise. In doing so, we identify key considerations and provide actionable recommendations for simulation programme design that is sensitive to diverse learners’ and all stakeholders’ needs.

Simulation-based education programme design involves many interrelated decisions that shape the creation and structure of experiences that are fit for purpose [3]. Simulation is widely used in pre-registration healthcare training because of the ability to replicate aspects of reality whilst controlling and manipulating factors to achieve learning outcomes [3, 4]. Specifically, the ability to control, manipulate, pause, and reset aspects of scenarios enables practice in a safe learning environment focused on learners rather than clinical imperatives [5]. Meanwhile, replicating aspects of real-world scenarios and contexts retains a degree of complexity and may assist in transferring learning to practice environments. Simulation activities are engaging and motivating, albeit at times stressful in their realism and cognitive load [6–8]. With a multitude of applications, however, designs need to consider purpose, learning outcomes and learning theories, and curriculum integration [2, 5, 9, 10].

Well-designed simulation-based education can supplement coursework to prepare students for and potentially (partially) replace early placements [11]. The CF-SCP was developed to guide the design of simulated clinical placements, as opposed to simulation-based approaches for skill development [2]. In summary, the CF-SCP identifies three components informing the design of simulation clinical placements (elements of practice education, quality criteria, and learning theory), and interconnected learning activities and processes that form the simulated clinical placement experience [2]. The authentic and workplace learning theories in the CF-SCP [2] are underpinned by constructivism [1]. Applied to preparing allied health students for placement learning, simulation learning outcomes need to include professionalism, learner attributes such as a willingness to learn, awareness of their own limitations and sense of self-efficacy, and communication and interpersonal skills [12–14], all of which can be facilitated in simulation [13]. In particular, grading and scaffolding challenges during simulation increase students’ confidence in realistic scenarios, preparing them to competently apply integrated knowledge and skills in complex workplace environments [15]. Simulation-based education also provides interprofessional learning opportunities that can be scheduled and manipulated to optimise learning [4, 5].

Simulation-based education must enable the transfer of learning by reflecting relevant aspects of reality [2] whilst balancing the emotional and cognitive load for the learner, all within resource limitations [16, 17]. It is critical to establish a safe context to learn through experience and manage challenging emotions, such as anxiety and disappointment, in the presence of others and without jeopardising positive regard [16, 18]. Simulation-based education must, therefore, include adequate orientation, pre-briefing, feedback, and debriefing, all with appropriate resourcing and facilitator training [5, 16].

Simulation-based education design must also consider learners’ diverse needs to ensure psychological safety and enable quality learning [5]. Realistic human patient simulation is particularly effective in enhancing skills such as communication and teamwork [4, 5, 19]. However, novice students are frequently adjusting to unfamiliar healthcare situations and environments. Further, patient interactions are inherently demanding, requiring competence in vocabulary and language, pronunciation and accent, humour, and interpersonal interactions, all of which are culturally based [20]. Reserved or anxious students may find the performative aspects of simulation challenging, while linguistically diverse students may become fatigued processing language for extended periods. Culturally diverse students may also need to manage acculturation in the context while simultaneously learning the knowledge and skills of their profession and their academic proficiency skills may, therefore, take longer to develop [21], particularly where terminology and complex interactions with patients are introduced early in the curriculum.

It is unsurprising then that, without appropriate support, international students are at a higher risk of failing in placement [22, 23] and can have difficulties where learning is constructed within a group of culturally dominant peers [24, 25]. Cultural and linguistic difficulties are often challenging for practice educators to address while sustaining patient care, and specific, staged, pre-placement preparation is recommended [20, 26]. Simulation can be designed to provide a safe psychological space that allows the student to learn from educators, academics, simulated participants and peers in an environment where time and tasks can be controlled to facilitate development. Situated in the dominant culture, simulation-based education also replicates local cultural expectations [27]. Explicit consideration and application of learning theory and simulation design will provide meaningful and effective learning experiences for a diverse range of students [1].

While the literature indicates that simulation-based education has a positive role in preparing students for allied health placements, few studies adequately describe the theoretical frameworks and factors informing the design of simulation for this purpose [1]. The CF-SCP provides a guide to simulation design based on learning theory, published evidence, experience and expert opinion [2]. While constructivism and workplace learning theories provide broad support for the foundations of simulated clinical placement design, it is important to consider the framework’s implementation with different learner groups and in interprofessional activities. It is important to consider the simultaneous perspectives of learners, simulated patients and practice educators [1, 2, 28]. Thoughtful consideration of these factors in the application of a conceptual framework extends the evidence base for the design of effective simulation-based educational approaches and enhanced preparation of students for placements.

This study aimed to identify implications for the design of allied health placement simulation from participants’ experiences of a simulation-based, interprofessional, novice placement preparation programme. Specifically, it was guided by the research question, what implications for the design of allied health placement simulation can be drawn from domestic and international students, practice educators’, and simulated participants’ experiences of an immersive foundational clinical skills simulation programme for interprofessional allied health students?

## Methods

This study was underpinned by a pragmatist philosophy and used qualitative approaches aligned with the value placed on the engagement of diverse stakeholders and the identification of actionable recommendations while acknowledging the situated and constructed nature of knowledge [29]. Specifically, multiple focus groups obtained participants’ perspectives, which were then examined using inductive reflexive thematic analysis [30] with reference to the aim of identifying the implications participants’ experiences had for the design of simulation. The findings were then abductively related to the body of literature, particularly the CF-SCP that informed the original simulation programme design, which in turn draws on authentic and early career workplace learning theories [2]. This combined use of pragmatist philosophy, a conceptual framework for simulation design, and reference to the literature guided the generation of actionable recommendations with broader transferability and applicability consistent with our research aim.

Ethical approval was provided by the Human Research Ethics Committee of the host institution (Approval No. 64382). All study participants gave voluntary and informed written and verbal consent to participate in the focus groups.

### Participants and recruitment

Simulation programme participants. All first-year pre-registration (bachelor’s and master’s) occupational therapy, physiotherapy, and podiatry students in the 2020–2021 academic year (*n* = 133) were invited to volunteer for one of up to 30 places in a pilot simulation programme. All eligible students were sent an email from an individual not associated with their course (LR), and volunteers completed and returned a consent and demographics form by return email. For equity, all students had access to an online learning course to support similar learning outcomes [28].

Study participant recruitment. Students completing the pilot simulation programme (*n* = 29) were invited into the research study by an individual not involved in their courses (LR). Study volunteers were offered a £15 voucher as a token of gratitude for their involvement, irrespective of whether they completed the focus group. All simulated participants (*n* = 12) and external occupational therapy and physiotherapy practice educators (*n* = 6) involved in the simulation programme were also invited. The sole podiatry educator held a dual role as an academic and practice educator and contributed indirectly to the study as a research team member.

### Simulation programme

The 1-week in-person simulation-based education programme was developed for occupational therapy, physiotherapy, and podiatry students at the end of their first year of study and immediately before their first placement. The programme aimed to provide students with the opportunity to rehearse their non-technical skills, explore the supervisory process with a practice educator in a safe environment, and begin to use clinical reasoning to apply their foundational knowledge to a clinical scenario with guidance from practice educators and academics. The programme was developed using principles of simulation-based education and Chu and colleagues’ [2] conceptual framework for simulated clinical placements (CF-SCP), carefully considering the practice context, the practice process, and the level of the students. The programme’s key elements are further described in Additional file 1.

Academics and practice educators collaboratively developed three authentic simulation scenarios, each suited for all three professions to achieve interprofessional learning outcomes while retaining relevance to a range of profession-specific placement settings. The scenarios represented frailty in a simulated acute hospital setting, head injury in a simulated home setting, and autism spectrum disorder complicating a musculoskeletal condition in a simulated community outpatient setting. The engagement with practice educators in the development phase ensured scenarios were authentic, immediately relevant to practice, and appropriately complex. To provide realism, they were implemented in simulated environments with simulated patients and family members (termed simulated participants; SPs) and practice educators supporting the students’ learning.

On the first day, the interprofessional group was introduced to the programme, including orientation, placement expectations, and the supervisory process. Students also practised giving and receiving feedback. Over the next 3 days, small profession-specific groups of students rotated through each of three different scenarios, delivered using a fishbowl approach. In this approach, student pairs take turns leading different aspects of interactions with SPs whilst their peers and practice educator observe and offer support. On the final day, students were reallocated into interprofessional groups, each participating in a multidisciplinary case conference related to one scenario from the week and exchanging feedback.

### Focus groups

Separate online focus groups for SPs, domestic-enrolled students, and international students were held using Microsoft Teams. Each focus group was scheduled by polling study volunteers. Focus groups were conducted within 3 weeks of the conclusion of the simulation programme while the students were on their first placement. The practice educator focus group took place in person on the final day of the simulation programme.

The four separate focus groups allowed each stakeholder group to speak freely from their own perspectives. The focus groups were semi-structured, and discussion was facilitated using an interview schedule developed for each stakeholder group (see Additional file 2). The interview schedules prompted discussions in each group addressing preparation for the programme, how the programme prepared students for placement (including specific positives and challenges), experiences of the programme, and recommendations. Each focus group was conducted by the same facilitator (LR) and lasted 80–110 min (average 1.5 h). No further focus groups were scheduled with remaining volunteers since the demographics of participants in the first four focus groups were broadly reflective of participants in the simulation programme and the insights offered within and across the groups loosely corresponded, suggesting the groups were addressing the most relevant information. The focus groups were professionally transcribed verbatim, including significant non-verbal communication (e.g. laughing or nodding) picked up from the audiovisual recording for the online groups.

### Data analysis

Data were inductively analysed using a reflexive thematic analysis approach to interrogate and interpret the participants’ statements and interactions to form relevant insights [30]. Data were analysed from the perspectives of one stakeholder group (i.e. one focus group) at a time, additively across the study. Two researchers (JB and LR) initially immersed themselves in the data by reading and re-reading the focus group transcript until it was broadly familiar, enabling them to engage with the ideas within. Having familiarised themselves with the first transcript, the researchers each created an initial set of codes interpreting how key ideas in the data related to the research question. Then, they coded the transcript, supplementing the code set to capture additional ideas until all ideas relevant to the research question were coded. After coding the whole of the first focus group, the two researchers met, discussed their independently created sets of codes, and developed an agreed framework of codes. Considering the research question, the limited number of voices from each stakeholder group, and the diversity between groups explored in the study, the consensus was to code ideas broadly. The two researchers then independently re-coded the first transcript using the agreed codes before meeting again to discuss any uncertainties and agreement across the transcript. From this, they created a shared document of codes and their scope to reflect the consensus on the interpretations and relevance to the research question they had reached.

The three remaining transcripts were coded independently using the agreed codes, with the researchers meeting between transcripts to compare coding and resolve any uncertainty in interpretation. Additional codes interpreting participants’ perspectives relevant to the research question were agreed upon during these meetings and added to the shared coding framework as relevant.

When all four transcripts had been coded using the agreed framework, JB and LR read through the definitions and coded data several times independently, noting down patterns and areas of overlap within the data. LR drew six central themes from the data, which were then clarified with the notes made by JB. A third researcher, DT, also considered these themes alongside the coding framework and contributed to their further elucidation. LR summarised each theme, and LR and JB tabulated representative quotes encapsulating each theme’s core tenets and breadth for the broader team.

Throughout the coding process, the framework of codes and coded data were reviewed in regular whole-of-team team meetings that referenced the design intent of the simulation programme, including against the CF-SCP, as well as the team members’ clinical, curriculum, and simulation perspectives to arrive at the key topics and actionable recommendations with broad applicability. As a final step, LR and JB reviewed the six themes, including findings and quotes, against the CF-SCP that had informed the original simulation programme design.

### Researcher positioning and reflexivity

Our pragmatist approach acknowledges the influence of the researchers’ positioning from inception through to dissemination of the research. In this case, the aim of the research was a central consideration in constructing a team of researchers and educators. The team included expertise in the design and delivery of simulation (DT, JB, and BJ), curricula (RT, KM, and BJ) and clinical education (DT, RT and EC) across the multiple professions, and international student health professions education (KM and JB), as well as a researcher independent of the simulation programme design and delivery (LR). Particular attention was paid to positioning and reflexivity for the team members central to the data collection, analysis, and reporting processes. Considerations included that LR engaged in debriefing and supervision throughout the data collection, drawing on the research and simulation expertise of JB, DT, and RT. Throughout the data analysis, JB and LR also each maintained reflexive logs. They acknowledged and discussed how they interpreted the data, considering they were external to the simulation programme delivery and had complementary experience in different universities, but were both occupational therapists and Anglo researchers in an Anglo research team. They engaged in regular whole-of-team consultations during the coding process, drawing on the specific expertise of each team member to review and clarify their interpretations. DT was also specifically engaged to further elucidate the themes as a simulation programme designer and physiotherapist. BJ was closely involved in discussions regarding the interpretation and writing as a simulation expert and external to the programme delivery. Throughout, the team process and reference to the project aim and conceptual framework ensured continued critical appraisal of the influences of team members’ roles in the project delivery and other experiences.

## Results

In total, 12 of the 29 students (9 females, 3 males) completing the simulation programme participated in focus groups: 7 of 18 domestic-enrolled students and 5 of 13 international-enrolled students. The focus group participants broadly represented the simulation programme participants’ gender, age, programme discipline, prior clinical experience, and ethnicity.

The six external practice educators (four females, two males) all participated in their focus group, with an equal split between physiotherapists and occupational therapists. All were local clinicians with prior experience supervising student placements. Four of the 12 SPs (1 male, 3 females) involved across the week participated in the focus group.

The final coding framework described 10 broad code areas referenced in participants’ experiences, each with specific codes and quotations. Considering the patterns and areas of overlap in the data, the team generated six themes representing the data with implications for the design of placement simulation: (i) engaging learning environment, (ii) realism and relevance, (iii) student confidence and communication, (iv) international students’ needs, (v) recommendations to facilitate further preparation for placement, and (vi) the importance of preparation to engage in simulation. Each theme is described below, with illustrative quotes in Table 1, one row for each theme or sub-theme in turn. For anonymity, each study participant is assigned a number within their focus group where FP1 was the first female participant introduced in that focus group, MP1 was the first male participant introduced, MP2 was the second, and so forth.

**Table 1:** Illustrative quotes

Theme	Quotes from Students	Quotes from practice educators	Quotes from simulated patients
(i) **Engaging learning environment: Part I-participant engagement and commitment**	International students: MP1: The [educators’] feedback would be more convincing; like I would trust their feedback and I would learn from their feedback Domestic students: FP2: We’ve barely been on campus, and I just wanted to be in person to get that confidence before actually being with a real patient	FP2: I knew there were learning outcomes and knowing when to try and let some of those pan out … and how much to push them versus doing what I would do as a clinical educator FP3: It felt really positive … the students were a real credit to themselves. They really engaged and obviously it sounds as though it was a programme that the people who wanted to do it signed up for it	MP1: It’s like anything you do the more you put into it before you start the more you are going to get out of it FP2: They [students] all understood why they were there and then got stuck in almost immediately
(i) **Engaging learning environment: Part II—enabling learning activities**	International students: FP1: We were really pushed to kind of figure it out on our own and at the time I was like ‘oh my God please help me, this is awful.’ But then actually looking back now I think that was the best way FP2: I found it useful to observe other people … because sometimes I’m not actually jotting down notes about the patients’ information, I’m jotting down how to ask the question instead Domestic students: FP4: having the case studies the night before was really helpful … I felt a bit more prepared MP2: the thing I liked most about the timeout was being able to step out of the situation and get immediate feedback … and then you’d be able to go straight back into it and almost rewind the scenario and start again	MP1: I like the timeout thing, it seemed to make it a bit more safe … you could ask them how it felt in the moment and you could ask their peers that were watching to give some feedback … I really liked that because when I struggle with students on placement, it’s the ones that don’t have that ability to reflect and communicate how they are feeling, are the ones that struggle … and that’s hard to teach. I find that hard to teach on placement. So, I think that was a real plus	FP2: Anytime they felt uncomfortable they could time out and talk through the scenario. I feel like the support they had from their classmates was very much appreciated
(ii) **Realism and relevance**	International students: FP4: During placement I got rejected by a few of the patients and because I’m on a simulation programme being told that … some patients might not want you to ask them questions or do anything to them and my supervisor thought I would be offended but I’m not because I already expect it FP1: The frailty one; I’m in an acute setting so literally all of my patients are exactly like that … I had a patient exactly the same as the one that we saw and I was like, ‘wow I know what to do’ or if a patient is like ‘oh I can’t hear you’ in the simulation … we learnt techniques to move past that FP1: I agree with the others I think personally one week is enough just like a little prep before placement, but I don’t think it should replace it Domestic students: MP1: It demonstrates that how effective it is because it felt quite real MP1: In placement … the patients were quite similar … it prepared me for what the client would expect and how you would approach an OT intervention and process … It gave me an idea of what to look for … One of the clients was almost identical in terms of problems and everything	FP2: We even had ward noise going on in our room, so it tried to make it a bit more authentic and I even found it distracting and I’m used to it … I think it’s a nice opportunity to give them lots of different experiences FP3: It added something foundationally from just how to act, how to adjust a bed, how to put somebody’s socks on and there were lots of questions that came out … ‘if I walk past this on the ward and it’s not a patient that I know what do I do?’ FP1: It would need to be extra rather than a replacement because what we’re generally seeing where I work as clinical educators it’s really hard to get everything in in that short space of time anyway FP3: It’s definitely going to be so valuable in supporting them to feel confident and get the most out of their placement, but … The MDT we did today, I’ve never known a doctor be as nice as me [laughs] … it provides the graded exposure, but it can’t be counted as exposure	FP3: You could see that it affected them because I got a bit tearful the first time, I could see the student that was talking to me, I could see her getting upset and that does affect the rest of the group so they do understand it better when they see somebody like that, it’s not a mannequin that sits there and doesn’t have any emotion. They have to realise that when they do anything like this people react differently MP1: I was absolutely as the patient, I was thinking if this person would come back weekly or whatever I have real confidence in my future now because there was this one person, I could really trust … They’re usually very subtle things they say and do … she was quite prepared to sit right next to me, hold my hand, do things with my hands: ‘Can you do this? Let’s move these’
(iii) **Student confidence and communication**	International students: MP1: It still boosts my confidence to perform better, to build a rapport with my client … I know I’ve got the ability to achieve my goals instead of expecting nothing FP4: I really learnt how to avoid specific language and just use simple words … I learnt so much … from my [domestic] students and also the clinical educators, which really helped me during my placement telephone consultations FP1: It helped my therapeutic use of self because that’s something in my supervision my practice educator commented on. She said I was really learning how to use my personality more and I’m more aware of my positioning … we got taught that like when FP2 did her bit … and those stuck with me that I took on to placement Domestic students: FP5: I would have been completely different if I hadn’t been able to speak to patients FP2: it’s made it much easier even just something as simple as … introducing yourself and what you do MP1: Overall in general with patients … It kind of got me out of an observational role into more of an active role	FP2: It just gives me so much hope for when they go into their careers and they are able to treat real patients with the same amount of empathy and understanding, it’s wonderful to see MP2: That was really valuable for them … that balance between you’ve got to strike a rapport with this person but then also maintaining a level of professionalism MP2: If you are a first-year student, the communication interaction is more important because the clinical skills depend on the area that you are working in	FP1: I’ve worked with the same group of students on both days, and they were a very different group on the second day … Even within the hour with the male student on the first day, having been quite weak to start with, he was much stronger by the end MP1: There was one girl … she was quite vocal … she wasn’t going to volunteer … but towards the end of the day, I think they’d run out of options, so she said, ‘oh I’ll go and do it then’. She was absolutely awesome, absolutely amazing because suddenly, I don’t know, I [the simulated patient] just clicked with her
(iv) **International students’ needs**	International students: FP2: Because we are not locals [laughs] … our mother tongue is not English so it’s very useful to observe how the locals really ask the questions FP2: My brain is so tired to process so many English and so much information [laughing] FP4: It’s really important that I have maybe a partner with me … someone to stand next to you and he or she can always jump in to help me if I am just stuck there FP2: It’s quite overwhelming so if I have a long lunch I can rest more and then I can perform better in the afternoon FP3: That was quite a long lecture in the morning so there was quite a lot for me to take in and it was just hard for me to digest	FP2: They put themselves forward and they’ve got the knowledge but again it’s the confidence and the ability to adapt MP2: One particular international student that spoke to me a lot I think it was almost like ‘if I could just practice this more, I’d feel more comfortable.’ MP1: One of the key learning objectives … is around communication … the international students need more of this FP3: We’re talking about communication and how intelligent and bright these students are and the potential that they have … it’s important to factor that in when making plans … practising what we preach and communicating with these students and seeing what do you feel you need rather than deciding for them	MP1: One girl from Hong Kong she volunteered to do a subjective assessment, but it was painfully hard for her to do. Once we timed out and had a discussion … it transpired that it’s just her culture would not allow her to talk in a friendly intimate way with a person of my age
(v) **Recommendations to facilitate further preparation for placement**	International students: FP1: [the assessment form] says for placement 1 … the lead comes from the practice educator… The simulation was independent … So it doesn’t match what we were expecting really … If we wanted to make it specifically tailored for P1 placement I definitely think that a supervisor actor should be included FP2: It would be more useful if we have a worksheet about the SOAP notes like maybe a sample FP1: Sticking to time because for example on the first day my educator spent nearly a full hour with one of the students and let them repeat … and then I didn’t get an opportunity to have a turn that day … that was the actual case that was most similar to the placement I’m on now Domestic students: MP2: Sometimes they’d [students] get stuck and wouldn’t know what to do and the educator would just, and like I said not all the time, but the educator would just give the answer. Whereas you may as well just come back to the group and ask if anyone else might know what to do	FP1: The students in our group were suggesting that it would be valuable to do it at different stages of their training so before each of their placements as they develop through their course, they’d be, probably be looking at slightly different things as they go through	
(vi) **The importance of preparation to engage in SIM**	International students: FP1: I actually felt the simulation was a bit last minute personally FP4: I kind of disagree because [name] told us a few weeks ago before and she said oh you’ll be having a simulation week practising your subjective and your objective skills for physio FP2: We know what we will be doing but we don’t know how it will be carried out, like how we would do it Domestic students: FP4: In the weeks leading up to it, it was quite uncertain what was happening purely because I think there was ethics approvals or something being waited for … But actually once we did find out it felt like, well I personally felt like I had a lot of information … I knew how the week was going to pan out which I really liked MP2: The simulations that we had earlier in the year probably made me feel a bit more ready to participate MP1: My roommate didn’t know much about it, I think he didn’t realise how useful it was or how good of an experience it would be so I think if it would have been explained better a lot more people would have actually signed up	MP2: It later revealed itself that [the case study] needs to be relevant for OT, physio and podiatry … someone with those needs is going to be super complex and make all the first years’ brains explode … it was fine in the end but I did have a concerns that all the OT stuff was going to be diluted … because of having to make it relevant for everyone MP1: University to write it [the case study] and then we can edit it FP × 3: Yes MP2: It seemed to rely quite heavily on how the students in that session responded … I felt like I wasn’t that prepared but actually in the moment it worked itself out FP3: It’s quite tricky to understand what the day is going to look like until you are doing it … I maybe would have appreciated having a real structure of what the days were going to look like and what was expected of us a little bit sooner FP4: I couldn’t make the educator training day but [name] sent me a recording of it … that was really valuable … I know [name]; we’ve worked together well that took some of the stress out FP1: So that is maybe something that needs to be clearer whether we should or shouldn’t [laughs] be working as the clinical educator	FP2: There was a lot of detail placed into how Rose acted to other people, the kind of body language that she portrayed … I very much based the character off those details rather than her diagnosis FP1: The first scenario where I was the daughter of the 83 year old lady, yes that was all very clear. I could also relate personally to that role in a way … The second day … My character there was very little information … we had a quick discussion beforehand and it was decided that I was going to be more negative … It was a much more difficult role for me because I had no background knowledge MP1: So if you do some research it just makes it more comfortable for you and you’ve got a better understanding of what the problems are that’s facing whoever you are acting FP3: I was OK because I can just react to how MP1 feels. I mean the information I had was fine, I didn’t really need that much

*FP* female participant, *MP* male participant

### Engaging learning environment

This theme featured two parts: participant engagement and commitment, and enabling learning activities.

*Participant engagement and commitment* were reflected in various ways through the study participants’ words. All stakeholder groups were invested in the programme and what they could gain from and/or offer to it. Students appreciated the commitment of the SPs and practice educators, and vice versa. Students were also enthusiastic and motivated by the opportunity, which facilitated their participation and learning throughout the programme.

*Enabling learning activities* was noted by all stakeholder groups to facilitate student learning and participation. Students described specific simulation programme features such as active engagement, observation of peers, preparation, and the ability to pause advanced their learning. The practice educators and SPs concurred, noting how they could support psychological safety and some skill development in simulation more effectively than on placement.

### Realism and relevance

Important to all stakeholder groups was that taking a turn in the simulation provided an immersive and convincing experience for students. Students took confidence from recognising that the scenarios and their simulation experiences were applicable to their subsequent placement, with some students able to directly implement their new skills. The SPs were cognisant of the realism of the scenarios in their portrayal, eliciting authentic responses from the students. Despite the realism and value, students and practice educators felt simulation should not replace placement exposure.

### Student confidence and communication

The students, practice educators, and SPs all noted that the students developed faith in their abilities throughout the week. Students and SPs alike reported how students increasingly volunteered their active participation and increased their comfort in interacting with the SPs across the week. Their active engagement contributed to their confidence in commencing the subsequent placement. The practice educators also felt that student interpersonal skills were commendable and believed communication should be a vital focus of the simulation programme in preparation for placement.

### International students’ needs

Practice educators expressed that international students may require different support than their domestic-enrolled peers in a simulation-based education setting. SPs reported on the cultural differences in communication, and practice educators observed that international students were less confident in taking turns. Some international students wished to take turns in pairs throughout the programme and described feeling tired from processing English for extensive periods. However, they found observing domestic students interacting with the SPs helpful. Notably, there were separate domestic-enrolled and international student focus groups, but the facilitator did not raise this topic. The domestic-enrolled students did not draw contrasts with their international student peers. Practice educators recommended consultation to ensure that the simulation environment is a place for international students to thrive.

### Recommendations to facilitate greater preparation for placement

Students used their experiences to suggest simulation design details they felt could better prepare them for their first placement. Suggestions included practice educators always being cognisant of the length of turns with SPs to share the opportunities and loads, and the programme providing additional guidance on documentation in Subjective Objective Assessment Plan (SOAP) format. Some students perceived that the expectations for student participation in the simulation were higher than in placement, and this might not have prepared students for interactions with practice educators on placement. Instead, for simulation to specifically reflect the first placement, these students suggested practice educators might interact within the scenario and directly with the SPs as they would on placement, although having opportunities for enhanced engagement of students was also recommended and aligned with other themes.

### The importance of preparation to engage in simulation-based education

The final theme relates to the specific details each stakeholder group noted that highlight the importance of preparation to engage in the simulation. Student perceptions around the information they received before the simulation varied; some thought the information was provided at the last minute, whereas others felt comfortable having the information they needed in a timely fashion. Importantly, though, some students felt that limited communication impacted uptake. For practice educators, the unclear understanding of the expectations of writing the scenarios was extensively discussed. It was ultimately widely agreed that the university should write scenarios and practice educators edit them. From the SPs’ perspectives, the details available in the scenarios to guide their representation varied between the case studies. Notably, study participants felt that the simulation programme came together well in practice. Although some practice educators and SPs perceived the uncertainty made things more difficult, none of the groups commented on the (lack of) preparedness of the other groups, indicative of and contributing to their commitment and engagement as outlined in the first theme.

## Discussion

This study aimed to identify implications for the design of allied health placement simulation from students’, practice educators’, and SPs’ experiences of a simulation-based interprofessional placement preparation programme. Key topics addressing the simulation design evident from this study are: designing for engagement and realism while balancing psychological safety and challenge factors, preparing all stakeholders for success, appreciating the needs of culturally diverse students, and balancing interprofessional and discipline-specific learning. Each of these will be discussed alongside the literature and with reference to the CF-SCP [2] before offering recommendations for designing an allied health placement simulation.

### Designing simulation for engagement and realism while balancing psychological safety

Simulation provides the opportunity to organise learning opportunities with scaffolding to meet specific learning outcomes, while also providing a level of authenticity to placement activities that support the transfer of learning into subsequent clinical placements [2]. In this interprofessional simulation programme, practice educators sometimes felt the tension in designing scenarios that balanced multiple student professionals’ authentic engagement against the complexity of the presentation. Their success was only known after the implementation when students reported confidence arising from recognising simulation scenario features in their placements. The students valued and respected the practice educators’ feedback given their authentic roles and perceived expertise [31], and this encouraged the students to respond to the challenge set. The students were, however, confronted at times when they perceived the expectations of their responsibilities within the safety of the simulation programme as more complex than the reality of their first placement. Although this was appreciated to enable learning and confidence for placement, consistent with other studies (e.g. [32]), the realism of the scenario evoked a strong sense of responsibility that challenged the ‘first-year student’ identity. This could be addressed through attention to briefing and debriefing the student and educator roles and the purposes of simulation in relation to clinical placements, as well as the learning outcomes [2]. Alternatively, the design of the simulation could lessen the exploitation of the safe environment as a place to experiment with challenging tasks and emphasise the authenticity of the practice educator role in the first placement, such as in the level of role modelling.

In designing simulation scenarios, psychological safety is paramount for learning [16, 33]. Simulation realism, particularly with SPs, can introduce performance nerves and anxiety as students strive to perform well for the patient, educators and peers [8]. Performance anxiety was noted in this study, although the presence of peers is an enabler in simulation [32]. In this case, peers provided a sense of support and community, as well as opportunities for modelling, which international students particularly appreciated. It was also apparent from all perspectives that students’ confidence to embrace challenge increased over time with repeated exposures to the same simulation style, demonstrating there would not be negative consequences of errors and illustrating the support of facilitators [16]. Although our findings indicate that psychological safety underpins and impacts the entire simulated clinical placement experience, the importance of this consideration is not clearly incorporated into the CF-SCP [2]. The presence of a committed practice educator contributed to the realism of representing a placement and supported the students’ input, considering the programme level and purpose [2].

### Preparing all stakeholders for success

Ensuring students, SPs, and practice educators are appropriately prepared for participation is key to establishing a safe and successful simulation programme. Students need preparation messages to which they can relate, from a person they trust, in a medium they are likely to access, and that provide practical guidance. In this study, some student cohorts had more time on campus or familiarity with simulation than others, influencing the programme uptake. Student participants also valued and recommended more specifically actionable details pre-simulation. At the orientation stage, preparing students for the challenges and growth opportunities of the simulation programme assists in establishing a safe learning environment [33, 34]. Some of these learner-related elements are addressed in the CF-SCP [2], but preparation and briefing are under-represented.

SPs in this kind of simulation programme portray a dynamic role in response to student actions while maintaining realism and relevance to the student learning outcomes. These study findings are consistent with the industry *Standards of Best Practice* [35, 36]. The emphasis on the case development domain of the SP *Standards of Best Practice* [36] reflects the complexity of this task and the extent to which it is programme-specific, in this case, a newly developed pilot. In contrast, other domains may have been better addressed (e.g. safety), more transferable across programmes (e.g. training), or less immediately evident to participants (e.g. programme management). This study highlights the time investment required for scenario development and the level of preparation required for SPs, which extends upon the CF-SCP’s focus on SP modalities from the perspective of affordances for learning [2].

Regarding practice educators, it is fundamental that they appreciate the learning outcomes and how to facilitate the learning activities to achieve these [37]. The need for formal training is noted, and there is considerable discussion of training for pre-briefing and especially debriefing simulation-based education [37, 38]. However, the evidence base to guide practice educators and their trainers in other aspects of facilitation is limited, particularly in interprofessional and allied health settings [38]. It is evident in this study that students and practice educators need to have a shared understanding of their roles and how these might differ in a simulation-based education setting compared to a placement setting. While this study solely addressed the initial implementation of a pilot programme, evaluating and revising the programme to create a sustainable model has been considered [28]. Clearly delineated roles and adequate training are paramount to initial buy-in and sustainability [39] and were noted by study participants as promoting their commitment to participation in and improvement through the programme. That is, well-prepared and committed practice educators and SPs provide students with respected models for their own professional learning behaviours. Similarly to the preparation of SPs, the focus of the CF-SCP is on facilitation and clinical supervision contributing to the authenticity of a simulated clinical placement experience and learning outcomes [2] and the preparation of practice educators might be further emphasised.

### Appreciating the needs of culturally diverse students

It is recognised that culturally diverse students may have different learning needs [27]. Differences are most evident in areas such as communication skills and learning preferences, however, it is apparent that learning needs are not clearly articulated by culturally diverse students or fully appreciated by educators [25, 40]. Culture influences help-seeking approaches and pervades interpersonal interactions, including between learners and between learners and simulation educators [27]. In this study, some international students found full-length simulation days tiring. While this may replicate and potentially prepare students for full-time placements, excessive fatigue may compromise learning, creating tension for programme design. As in the literature, study participants recommended recognising the tensions and directly involving culturally diverse students in generating solutions [41]. Further explication of learner characteristics and how they differ as sub-groups and individuals, rather than being a single entity as in the CF-SCP [2], is needed to prompt relevant simulation design considerations. Integrating culturally diverse students may benefit those students through the complex acculturation process and also advantage peers otherwise ignorant of cultural influences on healthcare and how to be responsive to underrepresented groups in healthcare [25]. While intercultural learning was not a core focus of this simulation, exposure to this across the curriculum emphasises the value health professions place on cultural competence [40]. Yet, there is a lack of guidance on how this might be achieved with allied health students and the input of students with lived experience is desirable.

### Balancing interprofessional and discipline-specific learning

Common student needs across the allied health disciplines in preparation to commence placements [12, 14] provide opportunities for common simulation design across professions [13] and for interprofessional learning. For example, while communication content may vary, building rapport, applying open and closed questions, probing for and clarifying information, addressing personal topics and emotional content, and managing the flow of an interview are core skills. Further, simulation is an effective strategy to develop communication and other placement readiness skills [13, 19]. In this study, the simulation programme and scenarios were developed to provide all students with opportunities to meet these communication objectives within the context of their own profession-specific content and learning outcomes. This provides efficiencies in developing scenarios and supporting resources and streamlines preparation for SPs. It is clear, however, that the complexity of scenarios involving multiple health professions must be managed to balance psychological safety and the level of challenge for students. It was also evident that practice educators with limited experience in simulation scenario design found developing scenarios with this balance challenging. Academics with specific simulation expertise are best suited to design the scenarios [9], guided by the CF-SCP considerations [2], and utilising the expertise of practice educators to ensure the relevance of the clinical situation to placements.

It is recommended that interprofessional learning opportunities are scaffolded across curricula and that simulation-based education may provide some opportunities [5, 42]. Bringing the students together in extended sessions at the beginning and end of this programme provided explicit interprofessional learning opportunities. However, there was little focus group discussion of the students’ interprofessional learning from the simulated multidisciplinary team meeting. This may have reflected the limited time devoted to interprofessional activity or lower engagement than might have been achieved with active collaboration [43]. It is also possible that when students were challenged to develop their knowledge and skills in readiness for their upcoming placements, they prioritised profession-specific development. The barriers to implementing interprofessional learning during novice placements [44, 45] may also have limited transfer of students learning from the simulation programme and, therefore, ready recall of this learning. Explicit pre-briefing and debriefing may prove especially important for interprofessional learning [5]. Encouragingly, despite the limited reflection about the interprofessional nature of the program, students did report an increased awareness of their own professional identity through this experience.

## Recommendations for simulation programme design

Considering all the findings alongside the existing literature and with specific reference to the CF-SCP as a guide to the design of simulated placements, we have identified and presented in Table 2 recommendations for simulation programme design. Reviewing the CF-SCP specifically, we would recommend retaining the existing design considerations and experience elements that emphasise replicating relevant placement features with consideration to quality criteria and learning theory [2]. We would add that psychological safety is a critical consideration that should be highlighted and industry best practice standards may supplement quality criteria. In addition to the consideration of placement elements and affordances for learning [2], needs assessment recognising learner sub-groups and individual characteristics, as well as preparation, orientation and briefing to bridge all stakeholders from the design into the experience, is indicated.

**Table 2:** Recommendations for designing allied health placement simulation

Simulation design element	Recommendation
Overall programme design	Consider the profession-specific/interprofessional balance in scenarios and learning outcomes relevant to the placement and learner level Design explicitly to address specific placement expectations Identify learner needs considering sub-group and individual differences, including through direct engagement with learners
Scenario preparation	Allow ample time for interprofessional consultation Collaborate using the different expertise of academics, practice educators and SPs Consider the authenticity/complexity balance (real enough)
Pre-simulation participant preparation	Offer early practical guidance for learners to psychologically prepare relative to their prior experience Provide sufficient detail and invest time in SP preparation Ensure training for practice educators relevant to their roles and learning outcomes
Orientation and briefing	Brief the learner and educator roles and the purposes of simulation in relation to clinical placements Brief learners on challenge and growth opportunities in simulation Provide guidance regarding the profession-specific/interprofessional balance in the simulation experience
Participation	Engage practice educators’ authentic roles where learners appreciate their expertise Utilise peer support Offer repeated opportunities in the same simulation style Allow scope for flexibility for experienced educators to meet diverse learner needs
Debriefing	Be explicit with students regarding the purpose of the debrief and the utility of guided reflection with links to placement and ongoing development
Cyclical evaluation	Engage in iterative evaluation to inform future programme design

## Limitations

Despite using a conceptual framework and other measures, the transferability of these findings and recommendations to other contexts is limited by the small sample and specific features of the simulation programme. Further, the students self-selecting to participate in the simulation and focus groups, may not represent the general student population. Results should be interpreted with caution as self-selecting students may be particularly motivated to learn in this format and the viewpoints participants shared may not wholly represent those of non-participants. While student participants in the focus groups were representative of those in the programme, the programme itself was disproportionately attended by occupational therapy students. The lead data analysts in this study (JB and LR) are also occupational therapists and the CF-SCP was developed in occupational therapy. Conversely, the focus groups included few representatives from the smaller discipline of podiatry, although the educator perspective of this discipline was represented in the research team. Finally, for the purposes of the focus groups, international students were grouped together and as opposed to domestic-enrolled students without detailed nuance as to student backgrounds.

## Conclusions

Simulation-based education is increasingly being embedded in pre-registration allied health programmes with a growing body of evidence supporting its effectiveness to support learners’ to develop their preparedness for placement and foundational technical and non-technical skills. From students’, SPs’, and educators’ perspectives, we have identified recommendations for the design of allied health simulation. These recommendations supplement the limited evidence base on factors and considerations informing the design of allied health placement simulation and extend the CF-SCP. Educators may draw parallels from these recommendations to their own simulation programme design. Future work may also integrate greater stakeholder co-design in simulation-based education.

## Supplementary Information

Additional file 1: Simulation programme characteristics.

Additional file 2: Focus group interview schedule.

## Data Availability

The datasets generated and analysed during the current study are not publicly available as they contain information that could compromise research participant privacy/consent but are available from the corresponding author upon reasonable request.

## Abbreviations

FP: Female participant
MP: Male participant
SOAP: Subjective, Objective, Assessment, Plan
SPs: Simulated participants (simulated patients and family members)
