# Supplementary material for: Educational design insights for interprofessional immersive simulation to prepare allied health students for clinical placements
Source: Adv Simul (Lond). 2024 Nov 27;9:45. doi: 10.1186/s41077-024-00316-0 (PMC11603799; doi:10.1186/s41077-024-00316-0)
Supplement: Supplementary file 1 — Additional file 1: Simulation programme characteristics. [file 41077_2024_316_MOESM1_ESM.docx]

Additional file 1: Simulation programme characteristics

| **Programme characteristic** (1) | **Description** |
| --- | --- |
| **Instructional design** | Curriculum integration: The program was scheduled for the whole week prior to the students’ first ‘traditional’ clinical placement at the end of their first academic year of study. |
| **Simulation environment** | Location: The university clinical skills simulation rooms include a mock acute care ward, outpatient clinic, and simulated home with half-height walls to allow student viewing.  Equipment: Each setting contained appropriate equipment. The simulated hospital ward had a profile bed, walking frame, oxygen and suction equipment. The outpatient clinic contained a plinth, table and chairs. The residential flat had a lounge, bedroom, kitchen and bathroom.  External stimuli: Ward sounds were on a continuous play loop for the frailty case study that was situated in the simulated hospital ward |
| **Learning objectives** | By the end of the week students would be able to   - Identify their own learning goals, and consider their strengths and weaknesses - Demonstrate appropriate communication skills when working with their supervisor, simulated patients and relatives, and multi-disciplinary team - Conduct aspects of professional behaviour (e.g., asking for consent, writing clinical notes, making a follow-up appointment) - Demonstrate some basic therapeutic interventions/exercises/advice for the patient to undertake - Reflect on this learning experience and identify new learning objectives for clinical placement. |
| **Participant orientation** | Students: The first day of the programme included an introduction to the week with an interprofessional simulation in the afternoon that orientated students to the ‘fishbowl’ style of simulation with simulated patients and relatives.  Educators: Most practice educators were collaboratively involved in the scenario design, and all received training on conducting fishbowl simulation, the use of pauses and student substitutions, and the debriefing model (below), either in person or via a recording of the in-person session and provision of supporting learning materials.  Simulated patients: Experienced actors from a local simulation academy, who provide training in role portrayal and feedback provision, were recruited to represent each scenario. They were provided a scenario briefing including expectations of them and the programme, and characteristics of the individual and scenario they would portray. |
| **Simulation event/scenario** | Duration, timing, frequency and repetitions: Each simulation scenario (one per day for three days) included a pre-briefing, two simulated patient interactions separated by a debriefing, and a final debriefing. In each case the first simulated patient interaction was 45 minutes long and focussed on taking a subjective history, while the second simulated interaction with the same patient was 150 minutes long and focussed on objective assessment and management. Within these sessions, students could pause and ask questions of their peers or practice educator, and swap roles with their peers. During the second simulated patient interaction students were able to practice profession-specific techniques.  Grouping: Students worked through the first day orientation and content as an interprofessional group. Students were then divided into two groups of ten occupational therapy students, one group of six physiotherapy students, and one group of three podiatry students. For the final day multi-disciplinary team meeting, students were allocated to interprofessional groups comprised of four occupational therapy students, two physiotherapy students and one podiatry student.  Simulated patients and confederates: Actors were used to represent each patient as well as an anxious daughter of the frail elder, the wife of the head injured man, and the mother chaperoning the adolescent with autism spectrum disorder.  Facilitators: Each group of occupational therapy or physiotherapy students was supported throughout by an academic and a practice educator with experience relevant to the scenario. For the small group of podiatry students, an academic undertook both roles. The practice educators were known to academic staff and worked in settings where students regularly completed placements. Their role was to support students in simulation, increasing the realism of the scenario for students. The role of the academics was to reassure students and assist help them draw links to the content addressed in their first year of study.  Clinical variation, adaptability of intervention, and range of difficulty: The three scenarios were designed with relevance to the interprofessional learning outcomes and each of the three professions. They represented acute hospital, outpatient, and home visit settings of care, and presentations of frailty, head injury, and autism spectrum disorder complicating a musculoskeletal injury. The academics and practice educators collaborated to create scenarios that were plausible for a patient a student my encounter with their supervisor on their first placement.  Non-simulation interventions and adjuncts: The first day provided orientation to the programme and addressed content relating to placements and placement supervision in a group didactic format. Also on that day was an interactive session on giving and receiving feedback. Additionally, students were provided with short vignettes introducing patient centred communication portrayed by actors and facilitated by a content expert. They were able to opt in to participate in these vignettes and reflect on the experience. On the final day, students participated in a simulated multidisciplinary team meeting facilitated by a practice educator and supported by the academic staff. |
| **Simulation scenarios** | Clinical variation and difficulty: Each scenario was collaboratively designed with practice educator involvement to enable realism and suitability to all three professions while addressing a wide range of potential placement settings and targeting an appropriate level of challenge. The scenarios represented frailty (acute), head injury (home), and a musculoskeletal condition complicated by autism spectrum disorder (outpatient or mental health setting).  Adaptability: Being in small groups enabled students to take turns at being in the simulations across the week and to observe their peers’ performance.  Standards/assessment: The recently implemented Common Placement Assessment Form (2), which aligns closely with the programme learning outcomes, was used by students across all three professions to self-assess their progress during the week with each simulation  Actor and confederate roles: Actors represented the patient and a relative for each scenario. |
| **Feedback and pre/debriefing** | Timing and duration: Each scenario day commenced with a 30-minute briefing, with a 90-minute debriefing between the two simulated patient interactions, and a final 30-minute debriefing.  Source, facilitators and providers: The practice educator facilitated the provision of feedback and guidance to students as relevant in the briefings, during time outs throughout the scenarios, and particularly in the debriefing sessions. After students’ self-reflection, the actors provided feedback in neutral character, then peers offered their perspectives. Finally, the practice educators provided additional points and summarised.  Content and structure: The feedback and guidance were directed toward having students perform in each scenario as though actively engaged with a patient on their first placement. The practice educators had differing levels of experience of simulation-based education and so were directed to use the structured approach for consistency. The ‘SHARP’ debrief model was used consistently. SHARP consists of **s**etting learning objectives, considering **h**ow it went, **a**ddressing concerns, **r**eviewing learning points and **p**lanning ahead (3). This debriefing method was chosen as it is simple and easy for novice facilitators to follow. |

1. Cheng A, Kessler D, Mackinnon R, Chang TP, Nadkarni VM, Hunt EA, et al. Reporting guidelines for health care simulation research: Extensions to the CONSORT and STROBE statements. Simul Healthc J Soc Simul Healthc. 2016 Aug;11(4):238–48.

2. Chartered Society of Physiotherapy. Common placement assessment form [Internet]. Author; Available from: https://www.csp.org.uk/professional-clinical/practice-based-learning/cpaf

3. Imperial College of London. The London handbook of debriefing. London: National Health Service; 2012.
