# Supplementary material for: Educational design insights for interprofessional immersive simulation to prepare allied health students for clinical placements
Source: Adv Simul (Lond). 2024 Nov 27;9:45. doi: 10.1186/s41077-024-00316-0 (PMC11603799; doi:10.1186/s41077-024-00316-0)
Supplement: Supplementary file 2 — Additional file 2: Focus group interview schedule. [file 41077_2024_316_MOESM2_ESM.docx]

**Additional File 2: Focus Group Interview Schedule**

1. **For Students**

1. How prepared did you feel to participate in the simulation?

*Example prompts: Did you feel prepared? What helped you to feel prepared? Monday briefing on sim and expectations? Each day pre-briefs? What might have helped you to feel more prepared to participate?*

1. Why did you decide to participate in the simulated programme?

*Example prompts: How did you think that it was going to help you?*

1. How did you find participating in the simulation programme?

*Example prompts:* *How safe did you feel to make a mistake in the scenarios?* *Was there anything that you enjoyed or did not enjoy about the programme?*

1. How did you feel the simulation helped you to achieve your learning outcomes or goals?

*Example prompts: What specifically did you learn? How did the facilitator help you learn? Was there a part of the programme that you thought was particularly valuable?*

1. Can you think of anything that did not go so well with your learning during your participation in the programme?

*Example prompts: If so, what and why?*

1. How do you feel the simulated programme prepared you for your first placement?

*Example prompts: have you faced any challenges whilst on clinical placement? If so, how did the simulation programme prepare you to face those challenges?*

1. How do you feel that the simulated programme impacted your communication skills on placement?

*Example prompts: With patients and relatives/ Practice educators/ multidisciplinary team*

1. Do you think that any element of the simulated programme could be improved and helped you feel more ready for placement?

*Example prompts: If so, what and why?*

1. Is there anything else that you would like to add about the simulation programme and the effect on your current placement experience?
2. **For Simulated Participants**
3. How did you feel about the preparation to be involved in the simulation programme?

*Example prompts: Did the scripts contain enough and the right information to enable you well enough to act the case?*

1. Do you feel the simulation programme helped to prepare students for their first placement in clinical practice?

*Example prompts: How?*

1. How do you feel the students developed their patient-centred communication skills during their interactions with you?
2. Do you think anything in particular went well with the students’ learning?

*Example prompts:* *What do you think the students learnt? What was the most valuable part of the programme for them?*

1. Can you identify anything that did not go so well with the students’ learning during the programme?

*Example prompts: If so, what did not go so well? Were there any challenges that you experienced whilst participating in the SIM programme? Why did you find these things did not go so well/ were challenges?*

1. How did you personally find participating in the programme as an actor role player?
2. Can you think of any ways in which the programme could be improved?

*Example prompts: If so, what? Why?*

1. Is there anything else that you would like to add about your experience from the sessions that you attended?
2. **For Practice Educators**
3. Did you feel prepared for your role in this simulation programme?

*Example prompts: Why?*

1. Do you feel the simulation programme helped to prepare students for their first placement? How?

*Example prompts: if yes/no why?*

1. Do you think anything in particular went well with the students’ learning?

*Example prompts:* *What do you think the students learnt? What was the most valuable part of the programme for them? Do you think they achieved their learning goals?*

1. Can you identify anything that did not go so well with the students’ learning during the programme?

*Example prompts: If so, what did not go so well? Were there any challenges that you experienced whilst participating in the SIM programme? Why did you find these things did not go so well/ were challenges?*

1. How did you personally find participating in the programme as a practice educator?
2. Would you or other practice educators be prepared to take part in this again? *Example prompts: Why?*
3. Can you think of any ways in which the programme could be improved?

*Example prompts: If so, how? Why?*

1. This year the simulation programme for Occupational Therapists and Physiotherapists replaced their first week of placement 1, whereas for the Podiatrists, this was extra. What are your thoughts on how this could be used for placement preparation or replacement in the future?

*Example prompts: Why?*

1. Is there anything else that you would like to add about your experience from the sessions that you attended?
